# Period poverty and mental health implications among college-aged women in the United States

**DOI:** 10.1186/s12905-020-01149-5

**Published:** 2021-01-06

**Authors:** Lauren F. Cardoso, Anna M. Scolese, Alzahra Hamidaddin, Jhumka Gupta

**Affiliations:** 1 School of Social Policy and Practice, University of Pennsylvania, Philadelphia, PA USA; 2 Department of Global and Community Health, College of Health and Human Services, George Mason University, 4400 University Dr., Fairfax, VA 22030 USA

**Keywords:** Menstruation, Period poverty, Menstrual health, Sanitary products, Mental health

## Abstract

**Background:**

The purpose of this study is to examine the frequency of “period poverty,” or not being able to afford sanitary products, among university students, and associations with poor mental health.

**Methods:**

An online survey was conducted with a nationally-drawn sample (N = 471) of college-attending women to assess the association between period poverty and depression. Period poverty was measured via two questions designed for this study; depression was measured with the standard PHQ-9. Multivariable logistic regression was utilized for analysis.

**Results:**

Among our sample, 14.2% of women had experienced period poverty ever in the past-year; an additional 10% experienced it every month. Compared to those who had never experienced period poverty, adjusted analysis revealed that women with monthly past-year period poverty were the most likely to report moderate/severe depression (AOR = 2.34, 95% CI 1.09–4.99), followed by those who had experienced it ever in the past year (AOR = 1.83, 95% CI, 0.99–3.38).

**Conclusion:**

Many young women cannot afford menstrual health products to meet their monthly needs, and this may impact their mental well-being. Improved access to affordable menstrual products is needed to support these young women.

## Background

It is well established that meeting one’s basic needs—food, water, shelter—is the necessary foundation for health and well-being. Research indicates that the inability to meet these needs can affect individuals’ mental health [1, 2]. For instance, in a study of 2,870 mothers across 18 cities in the U.S., prevalence of major depression or generalized anxiety disorder increased with severity of food insecurity after controlling for factors such as income and past-year eviction [3]. This suggests that unmet basic needs, such as food and nutrition, can impact mental health above and beyond other hardships.

Menstrual hygiene is also considered a basic need, yet very little research has examined how unmet menstrual hygiene needs may impact mental health. Only recently, and after much effort by a small group of researchers and advocates, has menstrual health and hygiene been recognized as a pressing public health issue [4]. The unmet menstrual health needs of women and girls globally is vast, and includes the inability to access safe, clean facilities and affordable menstrual products [5]. The World Bank estimates that 500 million women and girls globally lack access to adequate facilities for menstrual hygiene management [6]. They also lack access to menstrual products. For example, over 80% of menstruating women and girls in Bangladesh use inadequate materials (e.g. old cloth) instead of hygienic products such as pads or tampons [7]. These unmet needs have important educational consequences for the lives of women and girls. In schools where girls lack access to hygienic facilities, menstruation can contribute to absenteeism or leaving school completely [8].

While the overwhelming majority of evidence and advocacy on unmet menstrual hygiene needs center on the experiences of women and girls in low- and middle-income countries, new research suggests that the experience of “period poverty,” or not being able to afford menstrual products, may be a common concern for low-income women in high-income countries. In a recent study with 183 low-income women in St. Louis, Missouri, 64% of participants reported being unable to afford menstrual products in the previous year [5]. One-third of women (n = 61) reported using other products as a result, including rags, toilet paper, and their children’s diapers. Aside from this study, public health research, and particularly epidemiologic data, on the experience of “period poverty” is scarce. Furthermore, even less is known about the mental health implications of period poverty. Given prior research documenting associations between other unmet needs and mental health, and the added shame and stigma associated with menstruation, an assessment of period poverty and how it relates to mental health is warranted.

An investigation of potential associations between period poverty and mental health is particularly important among college students. According to the 2018 American College Health Assessment, 63% of college students felt “overwhelming anxiety” in the previous 12 months. More than 1 in 10 (12.7%) had seriously considered suicide in that same time period [9]. Additionally, given that recent reports indicate that 35–50% of undergraduates may be food insecure, high levels of period poverty may also be present and thus research is warranted [10].

The current study aims to fill this critical gap by quantitatively examining, among a nationally drawn sample of young women enrolled in undergraduate education, (1) the frequency of self-reported experiences of past-year period poverty, and (2) the associations between past year period poverty and mental health.

## Methods

This study is part of a larger survey on women’s menstrual health, symptoms suggestive of endometriosis, and stigma. The online survey was conducted in April 2019 among a nationally-drawn sample of undergraduate women.

The sample was selected with the assistance of Qualtrics, a research firm specializing in online surveys and recruitment. Potential respondents likely to meet the inclusion criteria received an email introducing the study and its procedures. Interested participants read the written consent form online and provided informed consent before completing the 20-minute, online survey. The study received Institutional Review Board approval from George Mason University (# 1159779-6).

Participants met eligibility criteria if they were aged 18–24, reported a female sex at birth, and enrolled as a student in undergraduate education. A total of 515 respondents completed the survey. For this analysis, 471 respondents were included. Those who have never had a period (n = 4) or who hadn’t had a period in the past-year (n = 27) were excluded from analysis.

Period poverty was assessed by asking two questions, first: “In the past 12 months have you struggled to afford menstrual products (such as sanitary pads or tampons)?” Those who answered “yes” (vs. “no”) were asked a follow-up question, “Do you struggle to afford menstrual products every month?”. Responses were used to construct a three-level categorical variable: participants who reported experiencing period poverty every month; participants who reported experiencing period poverty in the past year, but not on a monthly basis; and those who reported never experiencing period poverty.

Respondents who reported any past-year period poverty or period poverty every month were also asked, “have you done any of the following because you did not have enough money to purchase menstrual products?” and could select any of the following options: Used other products (e.g., toilet paper, fabric) as menstrual products, Borrowed menstrual products (e.g., from friends, coworkers, strangers), Left a menstrual product in too long, Had to go without menstrual products, I have always had enough money to purchase menstrual products, Other. These measures were developed based on previous menstrual health research [5]. An additional file provides these questionnaire items (see Additional file 1).

The Patient Health Questionnaire (PHQ-9), a 9-item scale that has been well-validated and widely used with a variety of populations including young people, was used to assess depression [11]. Respondents were asked, “Over the past 2 weeks, how often have you been bothered by the following problems?” Nine “problems” were listed (for example, “poor appetite or overeating”) and responses included “not at all,” “several days,” “more than half the days,” “nearly every day.” Respondents received a total score ranging from 0 to 27. A dichotomous variable was constructed: those who had none or mild depression (scores 0–9), and those who had moderate to severe depression (scores 10–27).

Previous health research among college students informed our demographic variables [12]. Respondents were asked their age (numeric responses), relationship status (not in a relationship, in a relationship but not cohabitating, in a relationship and cohabitating), self-reported health status (excellent, very good, good, fair, poor, don’t know), where they live (on- or off-campus), and enrollment status (full- or part-time). They were also asked their race (White; Black; Hispanic or Latino/a; Asian or Pacific Islander; American Indian, Native Alaskan, Native Hawaiian; Biracial or Multiracial; Other), though this variable was collapsed to 4 categories (White, Black, Hispanic or Latino/a, Other). Country of origin (259 response options) was constructed as a dichotomous variable, differentiating between individuals born in the United States and individuals born elsewhere. Sexual orientation (asexual, bisexual, gay, lesbian, pansexual, queer, questioning, same gender loving, straight/heterosexual, other) was collapsed to a 2-category variable (heterosexual/straight, other). Finally, two variables, mother’s education and father’s education (response options: less than high school completed, high school diploma or equivalent, some college, vocational or trade school, bachelor’s degree, master’s degree, professional degree, doctorate, don’t know, not applicable), were combined to create a “first generation” college student variable. Respondents who did not have a parent with a college degree were considered “first generation”; respondents with at least one parent with a college degree or higher were not considered “first generation” [13].

Frequencies and means (where appropriate) were examined for all variables. Bivariate analysis using Pearson’s χ2, t-tests, and one-way analysis of variance assessed the relationship between demographic variables and period poverty, and demographic variables and depression. Logistic regressions assessed the relationship between period poverty and depression, controlling for sociodemographic variables. To preserve power, some covariates were dichotomized for regression analyses. Significance levels were set at 0.05. All analysis was conducted in Stata v14.1.

## Results

The mean age of our sample (N = 471) was 20.6 years. Two-thirds of our respondents reported their race as White (65.6%), while 16.8% reported being Black, 11.3% Hispanic/Latina, and 6.4% were another race. The majority of the sample (92.6%) was born in the US; slightly over four in ten (43.3%) were first generation college students. Sixty-five percent reported their sexual orientation as “straight/heterosexual.” Around 3 out of 4 respondents were enrolled full-time (76.7%) and lived off campus (79.0%). In terms of relationship status, 46.3% reported being single, 35.9% were in a relationship but not cohabitating, and 17.8% were in a relationship and cohabitating. One in five respondents (20.3%) rated their health as “excellent,” while 1 in 10 (9.4%) rated it as “fair/poor.”

Among our sample, 14.2% of women had experienced period poverty ever in the past-year; an additional 10.0% experienced it every month. As a result, women coped with their menstrual product needs in a variety of ways. The most frequently mentioned coping methods included borrowing products (72.8%), using other materials in lieu of menstrual products (52.6%), using pads or tampons for longer than suggested (48.3%), and going without any products (26.3%). Experiencing period poverty differed significantly by race (*p* = 0.038). Past-year period poverty was most frequently reported by Latina women (24.5%), followed by Black women (19%), White women (11.7%), and women of another race (10.0%). Experiencing period poverty every month was also most frequently reported by Latina women (17.0%), followed by women of another race (13.3%), White women (9.1%), and Black women (7.6%). Respondents who were born outside of the US (*p* = 0.003) also reported higher levels of period poverty (past-year: 20.0%, every month 25.7%) than young women born in the U.S. (past year: 13.8%, every month: 8.7%). Experiencing period poverty also varied significantly by family education (*p* = 0.005). One in five first generation college students (20.1%) reported past-year period poverty, an additional 10.3% reported monthly period poverty. One in ten non-first generation students experienced past-year (9.7%) and monthly (9.7%) period poverty. No significant difference was observed for other sociodemographic variables (displayed in Table 1 below).

**Table 1:** Bivariate associations between demographics and past-year period poverty, and depression

Demographic variables	Sample N(%)^a^	No period poverty n (%)^b^	Past-year any period poverty n(%)^b^	Past-year monthly period poverty N(%)^b^	*P* value	No/minimal depression^b^	Moderate/severe depression^b^	*P* value
Total sample (n [%])	471 (100.0)	357 (75.8)	67 (14.2)	47 (10.0)		243 (51.6)	228 (48.4)	
Age (mean = X)	20.6	20.5	20.5	20.5	0.891	20.6	20.5	0.503
Race								
White	309 (65.6)	79.3	11.7	9.1	0.038	53.7	46.3	0.404
Black	79 (16.8)	73.4	19.0	7.6		46.8	53.2	
Latino/a	53 (11.3)	58.5	24.5	17.0		52.8	47.2	
Other	30 (6.4)	76.7	10.0	13.3		40.0	60.0	
Country of origin								
US	436 (92.6)	77.5	13.8	8.7	0.002	52.8	47.3	0.075
Other	35 (7.4)	54.3	20.0	25.7		37.1	62.9	
Sexual orientation								
Heterosexual/straight	306 (65.0)	78.1	13.4	8.5	0.228	60.1	39.9	< 0.001
Other	165 (35.0)	71.5	15.8	12.7		35.8	64.2	
First generation college								
Yes	204 (43.3)	69.6	20.1	10.3	0.005	49.0	51.0	0.329
No	267 (56.7)	80.5	9.7	9.7		53.6	46.4	
Enrollment status								
Full-time	361 (76.7)	77.0	12.7	10.3	0.246	52.4	47.7	0.549
Part-time	110 (23.3)	71.8	19.1	9.1		49.1	50.9	
Living situation								
Off campus	325 (79.0)	74.8	13.9	11.4	0.312	49.5	50.5	0.183
On campus	146 (31.0)	78.1	15.1	6.9		56.2	43.8	
Relationship status								
Single	218 (46.3)	78.0	11.9	10.1	0.107	54.1	45.9	0.383
Relationship, not cohabitating	169 (35.9)	78.1	14.8	7.1		51.2	48.5	
Relationship, cohabitating	84 (17.8)	65.5	19.1	15.5		45.2	54.8	
Self-rated Health status								
Excellent	87 (20.3)	80.5	8.1	11.5	0.184	57.5	42.5	< 0.001
Very good	169 (39.5)	76.9	16.0	7.1		61.5	38.5	
Good	132 (30.8)	81.8	9.9	8.3		45.5	54.6	
Fair/poor	40 (9.4)	67.5	22.5	10.0		25.0	75.0	

^a^Column percentages

^b^Row percentages

Just under half (48.4%) the sample reported moderate or severe depression. Depression severity only differed significantly by sexual orientation (*p* < 0.001), such that respondents who are not straight (vs. those who are) reported more severe depression, and by health status (*p* < 0.001). Those who had the lowest self-rated health reported the most severe depression.

Among women who reported experiencing period poverty every month, 68.1% reported symptoms consistent with moderate or severe depression, compared to 61.2% of women who had experienced any period poverty, and 43.4% of those who had not experienced period poverty. Unadjusted analyses in Table 2 (below) showed a significant relationship between any past-year period poverty and moderate/severe depression (OR = 2.05, 95% CI 1.20–3.50) and between experiencing period poverty every month and moderate/severe depression (OR = 2.78, 95% CI 1.45–5.31). These relationships remained significant in the adjusted analyses. In comparison to participants who reported no period poverty, young women who had experienced any past-year period poverty or monthly period poverty were significantly more likely to report moderate/severe depression (AOR = 1.83, 95% CI 0.99–3.38; AOR = 2.34, 95% CI 1.09–4.99, respectively).

**Table 2:** Unadjusted and adjusted logistic regressions for associations between past-year and past-month period poverty and depression (N = 471)

Experience of period poverty	None/minimal depression (%)	Moderate/severe depression (%)	Odds ratio (95% CI)	Adjusted odds ratio (95% CI)
No period poverty	56.6	43.4	Referent	Referent
Past-year any period poverty	38.8	61.2	2.05 (1.20–3.50)	1.83 (0/.99–3.38)
Past-year monthly period poverty	31.9	68.1	2.78 (1.45–5.31)	2.34 (1.09–4.99)

AORs controlling for age, race, first generation student, US born, living situation, relationship status, enrollment, sexual orientation, self-rated health status

## Post-hoc analysis

We conducted additional analysis to examine potential impacts of a prior mental health diagnosis on the association of interest in our study. Specifically, we ran an additional model where we accounted for prior self-reported mental health diagnosis (i.e., the respondent was asked if they had ever been told by a doctor, nurse, or other health professional if they had a depressive disorder, including depression, major depression, dysthymia, or minor depression). In this post-hoc analysis, the adjusted ORs were slightly attenuated and remained significant for the association between both any past-year period poverty and depressive symptoms (OR = 2.02, 95% CI 1.13–3.62) and monthly past-year period poverty and depressive symptoms (OR = 2.47, 95% CI 1.23–4.96). Future research is needed to examine any potential role that timing of diagnosis, lack of diagnosis, and medication may have on the association of interest.

## Discussion

In this nationally-drawn sample of college attending women in the United States, 14.2% had ever experienced period poverty in the past year; an additional 10.0% had experienced it every month. This is the first study to quantify unmet menstrual product needs among this population. This study also sought to assess the relationship between period poverty and depression. Findings revealed that period poverty was significantly associated with depression on a gradient. Compared to those who had never experienced period poverty, women who had experienced period poverty every month reported the most severe depression, followed by those who had ever experienced period poverty in the past year.

To our knowledge, there are no other studies that have examined the mental health impacts of period poverty, thus making comparisons difficult. However, our findings are broadly consistent with other research documenting an association between unmet basic needs and an elevated likelihood of poor mental health. For example, food insecurity has been found to be associated with depression among adults and depression, suicidal ideation, and anxiety among young adults and adolescents [14–16]. Similarly, people experiencing housing insecurity are more likely to report depression and anxiety than those who are stably housed [17, 18]. Notably, these studies collectively indicate that these unmet needs uniquely contribute to mental health, above and beyond the effects of income or wealth. The same may be true of unmet menstrual health needs, particularly given the shame and stigma associated with menstruation generally [19–21].

This study has several limitations. Income level is difficult to measure among college students via self-report, as discussed in existing research on food insecurity among university students [22]. Thus, this was not included as a covariate. Therefore, we could not test if period poverty was associated with depression independent of economic deprivation. Future research would be strengthened with the inclusion of appropriate economic measures, including food security, for this population. In addition, the sample was not probability-based and we do not have data on those who declined participation. Responses and findings are only generalizable to the demographics and experiences within this study. Finally, there is no standard way to assess period poverty. Although our measures were consistent with previous qualitative and quantitative research, more research is needed to validate our approach. Future research is also needed with broader populations of menstruators, including transgender and non-binary populations.

These limitations notwithstanding, this study is among the first to quantify period poverty and to examine its association with depression, and has implications for research and programming. Period poverty has not been definitively defined in academic spaces, and thus further scale development research is warranted.

Regarding programming and policy, addressing the mental health issues of college students is of particular concern given the high prevalence of stress, anxiety, and depression experienced by this population [9]. College administrators are addressing the issue by adding more comprehensive mental health services, but they should also examine the importance of unmet needs. Specifically, they should assess how period poverty may be contributing to the mental health issues among young women and consider making products more accessible. Some universities have such efforts underway—offering free menstrual products to students—and in some places in conjunction with efforts to reduce food insecurity.

More broadly, city- and state-level policy efforts to reduce the cost of menstrual products and increase their accessibility among vulnerable populations are gaining momentum. For example, as of 2016 New York City provides free menstrual products in public schools, homeless shelters, and prisons [23]. Similarly, starting in the fall of 2019, Boston began providing free menstrual products in public middle and high schools [24]. Several states have successfully repealed the “tampon tax” or the tax levied on menstrual products in states that consider them “luxury items” [25]. There have been added calls from advocates to remove other structural barriers to accessing menstrual products, such as requiring Medicaid and Supplemental Nutrition Assistance Program to cover menstrual products [26].

## Conclusion

Though preliminary, current study findings suggest that many young women cannot afford menstrual health products to meet their monthly needs, and this may impact their mental well-being. Improved access to affordable menstrual products is needed to support these young women. With additional research to help inform how period poverty impacts college students, there is tremendous opportunity to help address this critical, yet under-addressed issue.

## Supplementary Information

**Additional file 1** Period Poverty Questionnaire Items. This document includes the three questionnaire items related to period poverty that were used to assess period poverty prevalence in our study population.

## Data Availability

The datasets used and/or analyzed during the current study are available from the corresponding author on reasonable request.

## Abbreviations

AOR: Adjusted odds ratio
CI: Confidence interval
OR: Odds ratio
PHQ-9: The Patient Health Questionnaire
