# Supplementary material for: Period poverty and mental health implications among college-aged women in the United States
Source: BMC Womens Health. 2021 Jan 6;21:14. doi: 10.1186/s12905-020-01149-5 (PMC7788986; doi:10.1186/s12905-020-01149-5)
Supplement: Supplementary file 1 — Additional file 1 Period Poverty Questionnaire Items. This document includes the three questionnaire items related to period poverty that were used to assess period poverty prevalence in our study population. [file 12905_2020_1149_MOESM1_ESM.docx]

**Supplementary File 1: Period Poverty Questionnaire Items**

The following items were included in our questionnaire to assess period poverty prevalence in our study population:

In the past 12 months have you struggled to afford menstrual products (such as sanitary pads or tampons)?

1. Yes
2. No
3. Don’t Know

(If yes is selected) Do you struggle to afford menstrual products every month?

1. Yes
2. No
3. Don’t Know

Have you done any of the following because you did not have enough money to purchase menstrual products? (select all that apply):

1. Used other products (e.g, toilet paper, fabric) as menstrual products
2. Borrowed menstrual products (e.g., from friends, coworkers, strangers)
3. Left a menstrual product in too long
4. Had to go without menstrual products
5. I have always had enough money to purchase menstrual products
6. Other (please specify)
